# Development and validation of a robotic multifactorial fall-risk predictive model: A one-year prospective study in community-dwelling older adults

**DOI:** 10.1371/journal.pone.0234904

**Published:** 2020-06-25

**Authors:** Alberto Cella, Alice De Luca, Valentina Squeri, Sara Parodi, Francesco Vallone, Angela Giorgeschi, Barbara Senesi, Ekaterini Zigoura, Katerin Leslie Quispe Guerrero, Giacomo Siri, Lorenzo De Michieli, Jody Saglia, Carlo Sanfilippo, Alberto Pilotto

**Affiliations:** 1 Department of Geriatric Care, Orthogeriatrics and Rehabilitation, EO Galliera Hospital, Genova, Italy; 2 Movendo Technology Srl, Genova, Italy; 3 Italian Institute of Technology (IIT), Genova, Italy; 4 Department of Interdisciplinary Medicine, University of Bari, Bari, Italy; Singapore University of Technology and Design, SINGAPORE

## Abstract

**Background:**

Falls in the elderly are a major public health concern because of their high incidence, the involvement of many risk factors, the considerable post-fall morbidity and mortality, and the health-related and social costs. Given that many falls are preventable, the early identification of older adults at risk of falling is crucial in order to develop tailored interventions to prevent such falls. To date, however, the fall-risk assessment tools currently used in the elderly have not shown sufficiently high predictive validity to distinguish between subjects at high and low fall risk. Consequently, predicting the risk of falling remains an unsolved issue in geriatric medicine. This one-year prospective study aims to develop and validate, by means of a cross-validation method, a multifactorial fall-risk model based on clinical and robotic parameters in older adults.

**Methods:**

Community-dwelling subjects aged ≥ 65 years were enrolled. At the baseline, all subjects were evaluated for history of falling and number of drugs taken daily, and their gait and balance were evaluated by means of the Timed “Up & Go” test (TUG), Gait Speed (GS), Short Physical Performance Battery (SPPB) and Performance-Oriented Mobility Assessment (POMA). They also underwent robotic assessment by means of the *hunova* robotic device to evaluate the various components of balance. All subjects were followed up for one-year and the number of falls was recorded. The models that best predicted falls—on the basis of: i) only clinical parameters; ii) only robotic parameters; iii) clinical plus robotic parameters—were identified by means of a cross-validation method.

**Results:**

Of the 100 subjects initially enrolled, 96 (62 females, mean age 77.17±.49 years) completed the follow-up and were included. Within one year, 32 participants (33%) experienced at least one fall (“fallers”), while 64 (67%) did not (“non-fallers”). The best classifier model to emerge from cross-validated fall-risk estimation included eight clinical variables (age, sex, history of falling in the previous 12 months, TUG, Tinetti, SPPB, Low GS, number of drugs) and 20 robotic parameters, and displayed an area under the receiver operator characteristic (ROC) curve of 0.81 (95% CI: 0.72–0.90). Notably, the model that included only three of these clinical variables (age, history of falls and low GS) plus the robotic parameters showed similar accuracy (ROC AUC 0.80, 95% CI: 0.71–0.89). In comparison with the best classifier model that comprised only clinical parameters (ROC AUC: 0.67; 95% CI: 0.55–0.79), both models performed better in predicting fall risk, with an estimated Net Reclassification Improvement (NRI) of 0.30 and 0.31 (p = 0.02), respectively, and an estimated Integrated Discrimination Improvement (IDI) of 0.32 and 0.27 (p<0.001), respectively. The best model that comprised only robotic parameters (the 20 parameters identified in the final model) achieved a better performance than the clinical parameters alone, but worse than the combination of both clinical and robotic variables (ROC AUC: 0.73, 95% CI 0.63–0.83).

**Conclusion:**

A multifactorial fall-risk assessment that includes clinical and *hunova* robotic variables significantly improves the accuracy of predicting the risk of falling in community-dwelling older people. Our data suggest that combining clinical and robotic assessments can more accurately identify older people at high risk of falls, thereby enabling personalized fall-prevention interventions to be undertaken.

## Introduction

Falls are the second leading cause of accidental or unintentional injury and death worldwide [1]. In older people, falls are a major public health concern, as they frequently result in disability, impaired quality of life and excess mortality. In the USA, about 30% of people over the age of 65 experience a fall each year [2] and falls are the leading cause of injury-related morbidity and mortality among older adults [3, 4]. The estimated overall expenditures attributable to falls in the elderly already constitute a considerable health cost and are expected to increase further as the world population ages [5].

Many studies have demonstrated that several factors increase the risk of falling in older people [6], such as muscle weakness, vestibular dysfunctions, gait and balance impairment, neurological dysfunctions, visual and hearing problems, cognitive decline and orthostatic hypotension. Moreover, polypharmacy, depression and environmental or extrinsic factors have been indicated as co-factors, especially in older people [7].

The early identification of older adults who are at risk of falling is important in order to develop tailored interventions to prevent falls [3, 7]. To identify subjects at increased risk of future falls, studies have most commonly used a history of falls and the assessment of impairments in mobility, gait and balance [3]. Although systematic assessment of the risk of falls in the elderly is recommended [8], the tools currently used in geriatric medicine have not shown sufficiently high predictive validity in distinguishing between high and low fall risks [9]. Indeed, at least six major components are involved in maintaining postural control: constraints and biomechanical systems, movement strategies, sensory strategies, orientation in space, dynamic control, and cognitive processing [10].

Given that the commonly used functional balance tests have been developed simply to determine whether or not a patient has a balance problem and are unable to distinguish different types of balance deficits [11], more challenging measures are needed in order to better assess balance ability.

Very recently, the development of robotic devices capable of evaluating postural control has opened up interesting prospects for clinical research. *hunova* is a new robotic device that allows the evaluation of traditional stabilometric parameters and the implementation of various dynamic environments that stimulate postural responses. Owing to its accuracy, reproducibility and thoroughness in analyzing movement and postural control, which have already been shown in subjects with Parkinson’s disease [12] and elderly subjects [13], this robotic device could constitute an objective fall-risk assessment tool that may find clinical application in identifying and targeting individuals at high risk and in implementing specific training to rectify balance deficits.

Indeed, while the first step, which is specifically addressed in this study, is to identify people at risk of falls, the long-term goal of multidisciplinary evaluation should be to characterize the specific deficits related to the risk of falling, in order to address these deficits through personalized training.

The aim of this study was to develop and validate, by means of a cross- validation method, a multifactorial predictive model based on clinical and robotic parameters that could be applied in clinical settings to community-dwelling adults aged 65 years and older, in order to identify those at high risk of falling.

## Methods

### Participants and study design

Participants were recruited from the outpatient Department of Geriatric Care, Orthogeriatrics and Rehabilitation of Galliera Hospital (Genoa, Italy).

The study was approved by the Ethics Committee of the regional health authority (Comitato Etico Regionale (CER) Liguria) (reference number: 169REG2016) and was conducted in accordance with the ethical guidelines of the Declaration of Helsinki. All participants signed the informed consent form in accordance with these guidelines.

The inclusion criteria required that participants (both males and females) be ≥65 years old and have normal or slightly impaired cognitive function (at least 6/10 correct answers on the Short Portable Mental Status Questionnaire [14]). Participants underwent thorough clinical evaluation, in addition to a comprehensive geriatric assessment; they were excluded if they had speech and/or aphasia disorders, moderate-severe cognitive impairment or dementia, severe heart disease or respiratory failure, a degenerative neurological disease (e.g. Parkinson’s disease, multiple sclerosis), life expectancy less than six months, non-femoral bone fracture in the previous 6 months or femoral fracture in the 12 months prior to enrolment.

At the baseline, participants underwent clinical and robotic assessment, and were then prospectively followed up for 1 year. At the end of the baseline assessment, patients were instructed to record in a dedicated diary any falls, any clinical investigations performed as a result of falls or any hospital accesses due to falls. Falls were monitored by means of clinic visits or telephone calls. After 6 and 12 months, participants were contacted by phone and invited to undergo a follow-up clinical examination; if they could not come to our outpatient clinic, they were interviewed by phone. Phone interviews were semi-structured and were conducted by professional healthcare staff trained in fall assessment and telephone interview techniques. To minimize participant burden, the content of the calls was limited to whether the subject had been hospitalised during the period, the number of falls suffered in the period, and any resulting injuries that required medical and/or radiologic evaluations.

A fall was defined as “an unintentional change in position that results in a person coming to rest at a lower level or on the ground” [15]; falls due to syncope or extreme/unavoidable circumstances were excluded.

### Clinical assessment

At the baseline, information on age, sex, medical history and medications (# of drugs) was collected, and the number of falls in the previous year was recorded. Personal autonomy was assessed by applying the Barthel Index [16] and Instrumental Activities of Daily Living (IADL) [17], while the presence of comorbidity was ascertained by means of the Cumulative Illness Rating Scale (CIRS) [18].

Physical performance was evaluated by means of Tinetti’s Performance-Oriented Mobility Assessment (POMA) [19], the Timed Up & Go (TUG) [20], Gait Speed (GS) [21] and Short Physical Performance Battery (SPPB) tests [22]. Regarding gait speed, a threshold of 0.8 m/s was chosen and subjects were classified as under-/over-threshold (low GS) [23]. Baseline demographic and clinical characteristics of the study sample are reported in Table 1.

**Table 1:** Baseline demographic and clinical characteristics of the study sample.

Characteristics	All subjects (n = 96)	Fallers (n = 32)	Non-fallers (n = 64)	Significance (p value)
Age (years)	77.2±6.5	79.1±6.6	76.2±6.3	*0*.*050*
Gender				*0*.*416*
• Male	34 (35.4%)	10 (31.2%)	24 (37.5%)	
• Female	62 (64.6%)	22 (68.8%)	40 (62.5%)	
History of falling (§)	68 (70.8%)	28 (87.5%)	40 (62.5%)	*0*.*026*
BMI (Kg/m^2^)	26.44±4.56	26.81±5.38	26.34±4.17	*0*.*617*
Barthel Index	96.68±5.99	96.30±7.04	96.81±5.49	*0*.*701*
IADL	7.33±1.53	7.30±1.40	7.32±1.63	*0*.*954*
SPMSQ (correct answers)	9.64±0.75	9.64±0.70	9.63±0.79	*0*.*965*
CIRS severity	1.36±0.26	1.35±0.18	1.37±0.30	*0*.*757*
CIRS comorbidity	0.95±1.57	0.64±1.05	1.13±1.79	*0*.*493*
# of drugs	3.75±2.44	3.88±2.17	3.73±2.61	*0*.*775*
POMA	26.61±2.51	27.06±1.93	26.35±2.78	*0*.*197*
TUG (s)	9.63±3.23	9.84±2.57	9.63±3.53	*0*.*762*
Gait Speed (m/s)	1.04±0.27	0.95±0.22	1.08±0.28	*0*.*021*
SPPB	8.33±2.46	7.58±2.55	8.68±2.37	*0*.*039*

Values are expressed as mean ± standard deviation or number (%).

The unpaired t-test was used to compare means, while Fisher’s exact test was used for categorical data.

(§) ≥1 fall in the year before the baseline visit.

BMI: body mass index.

IADL: instrumental activities of daily living.

SPMSQ: short portable mental state questionnaire.

CIRS: cumulative illness rating scale.

POMA: performance-oriented mobility assessment.

TUG: timed up-and-go test.

SPPB: short physical performance battery.

### Robotic evaluation

**Fig 1:**
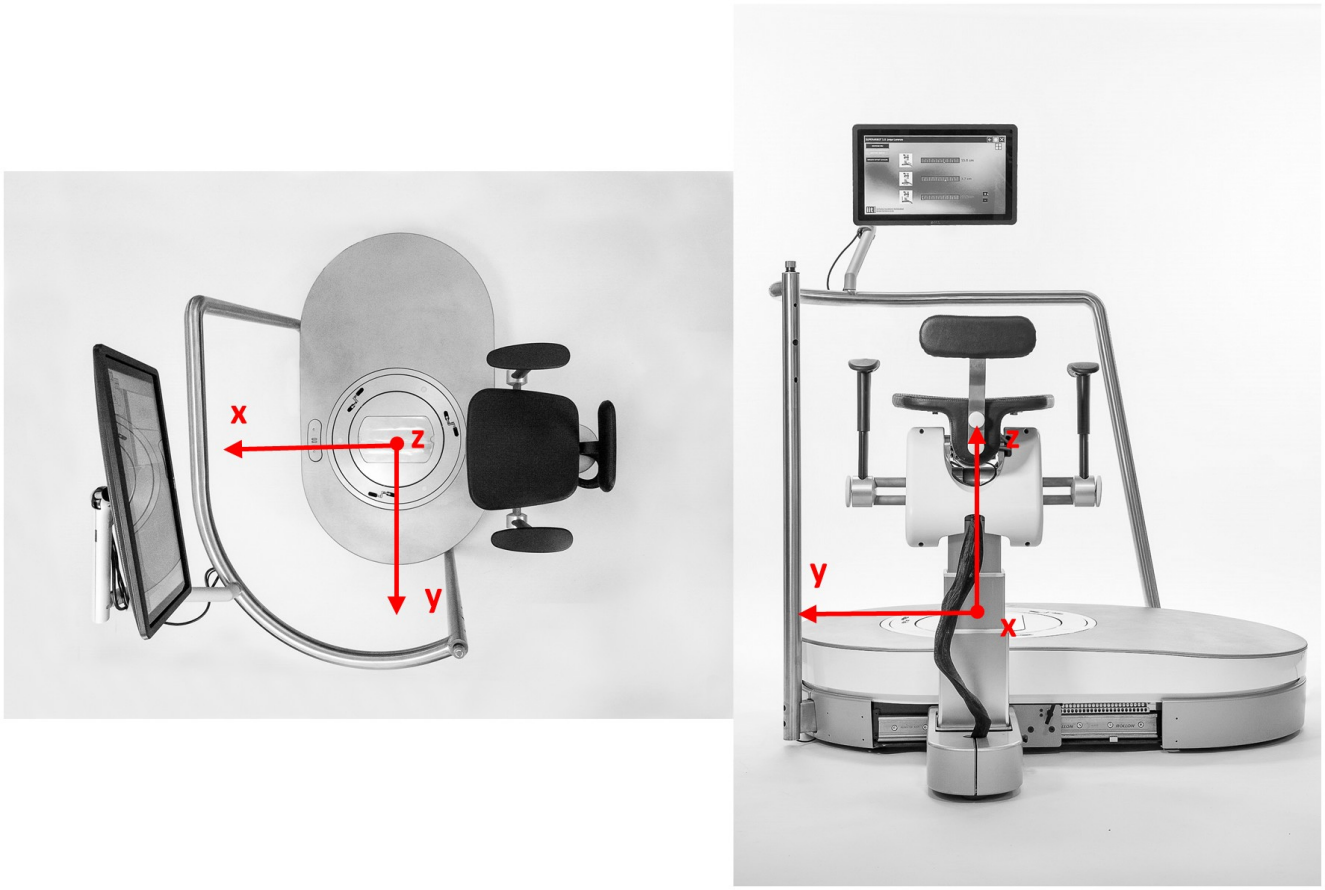
*hunova* robot. The *hunova* device is shown from above (a) and from behind (b).

Robotic tests were performed by means of *hunova* (Movendo Technology, Genoa, Italy, Fig 1) [24] a robotic medical device for the functional sensory-motor evaluation and rehabilitation of the ankle, lower limbs and trunk. The device consists of two electromechanical sensorized platforms, one positioned under the feet and the other positioned under the seat, which enable the subject to be assessed in both the standing and sitting positions. The device operates in conjunction with a wireless 9-axis sensor (Inertial Movement Unit—IMU, including accelerometer, gyroscope and magnetometer, which is a part of the device and is certified with the full system) located on the subject’s torso, to monitor trunk movements [24]. The robotic assessment consisted of the following 7 exercises. Tests were run in all patients in the same order (from Exercise 1 to Exercise 7), in a single session.

#### Limit Of Stability (LOS) test (Exercise 1)

While standing on the platform in static mode and wearing an inertial sensor (IMU) on the trunk, participants were asked to lean as far as they could in the directions indicated on the display screen (forward, backward, left and right). This test evaluated subjects’ ability to move the center of pressure (CoP) in anteroposterior or mediolateral directions within the base of support [25].

#### Balance tests in different conditions (Exercises 2 to 6)

Subjects were placed in the same position as in Exercise 1 and had to keep their balance for 20 seconds during unsupported standing when: a) the platform was static (with open eyes—Exercise 2 and with closed eyes—Exercise 3); b) when the platform was unstable, i.e. responding to the subject’s postural oscillations and sway/tilting movement along the x and y axes, with a maximum tilt of 10° in each direction (with open eyes only—Exercise 4); c) when the platform was moving along a default circular trajectory (with open eyes only—Exercise 5), and d) when the platform generated perturbations with impulses of 6 degrees in random directions (forward, left, right) and in random order (with eyes open only, Exercise 6). In Exercise 5 the platform tilted independently from the subject’s sway, following a pre-programmed angular trajectory obtained by using a position controller. Specifically, the platform moved according to the following equations:

$$\begin{array}{c} \theta_{ML}=Asin(2\pi ft)\\ \theta_{AP}=Asin(2\pi ft-\frac{\pi }{2}) \end{array}$$

where *θ*_*ML*_ and *θ*_*AP*_ are the angular tilt around the ML and AP axes, respectively; A is the maximum angular rotation (2.5°) an f is the frequency (0.2 Hz).

In exercise 6, the platform moved independently, in this case according to pre-programmed downward angular tilting along three different rotational axes: toes down, i.e. forward tilt of the platform along the y axis, right foot down i.e. rightward tilt around the x-axis, left foot down, leftward tilt rotation along the x-axis. The platform rotated according to a Gaussian profile trajectory with a peak of 6° at 330 ms after the onset of perturbation (mean velocity~16.5°/s). Each different perturbation was presented 3 times in random order. The time interval between consecutive perturbations was around 4.7±0.6 s, in order to avoid expectation or guessing, with a minimum value of 4 s to prevent the previous perturbation from influencing on the current one. This exercise included a familiarization phase, in which the subjects, while standing on the platform, experienced an example of how the platform could move during the actual test. Exercises 2, 3 and 4 provided information on subjects’ postural control in static and dynamic conditions by measuring spatial and temporal stabilometric parameters that described the trajectory followed by the CoP in the upright stance (Exercises 2 and 3) or during angular displacement of the platform (Exercise 4) and oscillations of the trunk. Exercises 5 and 6 tested the reactive postural control component of balance, i.e. the ability to recover stability after an external perturbation. In these tests, we extracted information on the compensatory strategies of the trunk in response to platform perturbations, namely oscillation of the upper part of the body [25].

#### Five Times Sit-To-Stand (FTSS) test (Exercise 7)

Subjects were instructed to stand up from a robotic chair five times as quickly as possible. The participants performed the task by starting and finishing in the seated position on the static *hunova* platform. The total duration of the FTSS test, the time required to stand up and sit down during each repetition, and the mean time required to stand up and sit down were calculated [26].

During evaluation, we positioned the handrail on both sides as a safety precaution; however, subjects were instructed to not touch the handrail unless they felt unsafe; if they touched the handrail, the trial was considered invalid and they had to repeat the test.

### Robotic data processing

Signals from the force-torque sensor, position sensor and trunk sensor were recorded at a sampling frequency of 30 Hz. The following formula was used to calculate the Center of Pressure (CoP) coordinates (CoPx, CoPy), at each recorded sample:

$$CoPx=\frac{Mx}{Fz},CoPy=\frac{My}{Fz}$$

where *Mx* is the torque in the mediolateral direction, *My* is the torque in the anteroposterior direction and *Fz* is the resultant force in the vertical direction, measured by the 6-axis force-torque sensor positioned under the center of the foot platform [27].

For Exercises 1–5, the parameter analysis considered the total duration of the task (80 seconds for Exercise 1 and 30 seconds for Exercise 2–5). For Exercise 6, we segmented the data in different epochs, from 0.25 seconds before the start of the perturbation to 1.5 seconds after the perturbation, and we focused the analysis from the start of the perturbation until 1 second after [12]. The beginning and end of each perturbation were detected by looking at the platform’s angular displacement signal.

In the LOS test (Exercise 1) we calculated the maximum CoP displacements (in cm) in each direction investigated by looking at the maximum shift of its coordinates (CoPx and CoPy) during the entire task [28, 29].

In the balance tests (Exercises 2–6) the following indicators, usually computed in standard posturography [30–32] and already validated for *hunova* for elderly subjects [13], for the trunk sensor and the platform (base or seat) were considered:

1. sway area (SA—[cm^2^]): the area of the 95% confidence ellipse of the statokinesigram of the CoP in the standing static condition for Exercises 2 and 3, and of the projection of the angular displacement of the platform in the standing dynamic unstable condition for Exercise 4. The 95% confidence ellipse can be defined as the surface that contains (with 95% probability) the individual points that make up the statokinesigram.
2. sway path (SP—[cm]): the length of the oscillation path of the CoP trajectory in the standing static condition (Exercises 2 and 3), and of the projection of the angular displacement of the platform (in standing dynamic unstable conditions, Exercise 4).
3. Anterior-Posterior and Medio-Lateral range of Oscillation (APO and MLO, respectively–[cm]) of the CoP (Exercises 2 and 3) in static conditions, or of the projection of the angular displacement of the platform in dynamic unstable conditions (Exercise 4). These parameters evaluate the extent of oscillation in the anterior-posterior or mediolateral direction and are proportional to the instability of the subject. They were computed by looking at the maximum and minimum shift of the CoP coordinates, i.e. CoPx (mediolateral range of oscillation) and CoPy (anteroposterior range of oscillation).
4. APO and MLO of the trunk ([deg]). These parameters provide information on the trunk compensation and trunk control strategies required to maintain balance. They are based on the trunk sensor signal (IMU sensor) and were computed by comparing the maximum and minimum degrees of inclination of roll (mediolateral range of oscillation, MLO) and pitch (anteroposterior range of oscillation, APO) angles. In the *random perturbating* condition (Exercise 6), the range of oscillation in the anteroposterior (APO) and medio-lateral (MLO) directions was computed after each perturbation.
5. trunk variability (VA—[m/s^2^]**)**: the standard deviation of trunk accelerations measured by the trunk sensor; this is a measure of the extent of movements of the trunk during the task, and was calculated for Exercises 2–5.
6. Oscillation time ([s]): the average time taken to stabilize the trunk in response to perturbations of the base. This was computed only for the random perturbating condition (Exercise 6), for each direction of perturbation (forward, left, right).
7. Anterior-Posterior and Medio-Lateral maximum oscillation of the trunk [deg]: the maximum oscillations (i.e. maximum angular displacement of the trunk) in the mediolateral (roll) and antero-posterior (pitch) directions were considered. These parameters were computed only for the random perturbating condition (Exercise 6); only the mean values between directions of perturbation (maximum tilt in ML direction and maximum tilt in AP direction) were considered in this analysis.

These indicators (a) to (g) are proportional to the subject’s instability: the greater the values, the lesser the subject’s ability to maintain balance [33–36].

In the FTSS test (Exercise 7), we calculated the total duration of the five sit-to-stand movements, the time needed to stand up and sit down during each repetition, and the mean time needed to stand up and sit down. To compute these parameters, we divided the task into the different phases of movement (i.e. sit-to-stand and stand-to-sit) by monitoring the force-torque sensors in the base and seat in conjunction with the IMU sensor. The sit-to-stand phase was deemed to have started when the forward inclination of the trunk exceeded a threshold of 15 degrees and to have ended when the subject’s load was completely on the base-platform, without trunk inclination (pitch<15°); similarly, the stand-to-sit phase began when the trunk started to incline forward (pitch>15°) and ended when the load recorded on the seat-platform was the same as that recorded at the beginning of the task (i.e. baseline in seated position) without trunk inclination (pitch<15°).

All the robotic parameters considered are reported in S1 Table.

### Data analysis and statistics

#### Experimental setting of training and validation of the model

The problem of fall-risk assessment was defined as a multivariate classification problem in a supervised classification setting in which each patient is a sample of the population and to each patient is assigned a set of input variables (clinical and robotic variables) and an output label, which indicates the risk category. The goal of this analysis is to identify a multivariate regression function for risk probability that has the highest possible predictive performance on an independent set of samples, i.e. a set of samples which have not been used to define the regression function.

Model performance was measured by means of the area under the receiver operator characteristic curve (AUC-ROC) and the 95% confidence interval (CI) as an overall index of diagnostic performance of our models [37]. For each model, we then calculated: mean precision (positive predictive value), sensitivity (recall, true positive rate), specificity (true negative rate), and Matthews correlation Coefficient (MCC). AUC-ROC values near to 1 indicate a higher probability of correct classification, whereas values near to 0 indicate a higher probability of incorrect classification. The positive predictive value was defined as the proportion of participants who were correctly classified as fallers by the algorithm. Sensitivity was defined as the ratio of the number of fallers correctly classified to the total number of fallers. Specificity was defined as the ratio of the number of non-fallers correctly classified to the total number of non-fallers. We used the MCC score as a measure of the quality of our classification.

Sensitivity, specificity and MCC were calculated for the cut-off that maximized the Youden index [38].

In order to define the parameters of the regression function, a supervised learning algorithm was trained on a training set for different values of the algorithm hyperparameters; the optimal value of the hyperparameters was selected by evaluating the performance on an independent validation set, and the estimated performance of the selected hyperparameters was evaluated on a further independent test set. In order to overcome the problem of the small sample size and to increase the stabilization of feature selection, *v*alidation and testing were performed by means of two nested loops of 5-fold cross-validation [39–41].

This *model validation* technique consists of randomly splitting the data into 5 equal ‘folds’, so that each set contains approximately the same percentage of samples of each target class as the complete set. Applied to the inner loop, this means that 4 of these folds are used to train the classifier (training set) and the remaining fold is then used to assess the performance of the classifier for different values of the hyperparameters (validation set); this is done for each possible combination of training and validation sets. Similarly, the outer loop is used for testing. Proceeding this way reduces the risk of overfitting and ensures robust estimates of the statistical summaries.

#### Selection of the best performance algorithm

The algorithmic framework described above was then used with different machine-learning algorithms and different subsets of input variables. A preliminary comparison of different algorithms on the subset of variables identified in previous studies (unpublished data) was performed. Linear models, such as Logistic Regression and Lasso and methods based on decision trees, such as Random Forest and Gradient Boosting, were compared. The best models appeared to be linear models, which are less prone to overfitting in settings in which the number of samples is limited in comparison with the number of input variables. As regards the algorithmic framework, we chose the Lasso method because: i) we needed a linear model to deal with the problem of dimensionality (the number of variables was of the same order of magnitude as the sample size), and the Lasso method has a lower degree of freedom than more complex models built on the same set of variables; ii) sparse methods, such as Lasso, provide a strong penalty term in the underlying regression problem, leading to more stable solutions even when the number of variables is of the same order of magnitude as the samples size [42]. Indeed, compared with logistic regression, the Lasso method is more likely to remove the less significant variables, and therefore to limit overfitting even when the number of examples is limited.

#### Identification of the best predictive model that included only clinical parameters: A priori selection

In order to identify the best-performing model that included only clinical variables, we first made an *a priori* selection of the main parameters commonly used in screening for the risk of falling [9, 11]. Different combinations of clinical variables were then tested in order to define the set of clinical assessments with the highest performance in fall-risk prediction. In this case, the selection of variables was based exclusively on *a priori* decision and was run in a single step, in which each group of variables was evaluated. Indeed, variable selection was based on our knowledge of clinical practice, i.e. we tested combinations of variables that could themselves constitute a clinical screening test for fall risk.

#### Identification of the best predictive model that included only robotic parameters

In order to identify the best-performing model that included only robotic variables, we made an *a priori* selection by considering different factors, such as: i) knowledge obtained from the literature and previous results; ii) the importance of each parameter, as indicated by significant differences between fallers and non-fallers.

#### Identification of the best predictive model that included clinical and robotic parameters: Model selection

The third step was to identify the best-performing model that included both clinical and robotic variables. This model selection was run in different steps, in which different configurations of variables were tested. Since we had to deal with a problem of dimensionality when we jointly considered clinical and robotic variables, we needed to incorporate prior knowledge, i.e. we discarded *a priori* those variables for which we could not collect any significant evidence in the literature or in our specific experience that was relevant to the problem under study. Thus, the features of each configuration tested were selected by considering various factors, such as: i) the importance of each parameter, as indicated by significant differences between fallers and non-fallers; ii) the importance of each parameter according to the literature, and iii) correlations among parameters. Moreover, the feature selection made at each step considered the results of the ROC AUC score in the previous step. By using these criteria, we selected a minimal dataset of clinical and robotic parameters, as follows:

- First, we identified a minimal set of clinical parameters by using a fixed dataset of robotic variables and comparing different configurations of clinical assessments. Specifically, the subset of robotic variables was selected on the basis of the results of a previous study [13], in which we investigated the correlation between *hunova* parameters during standing and sitting balance tasks and the Short Physical Performance Battery test (SPPB) in community-dwelling older adults; in that study, we found the strongest correlation with trunk control parameters in standing conditions (see the selected subset in S2 Table).
- To further improve the performance of the model, we then fixed the set of clinical parameters as the best set found in the previous step, and compared different configurations of robotic parameters.
- Finally, having fixed the data-set of robotic parameters to the best data-set identified in the previous step, we conducted a further analysis to test the best data-set of clinical parameters to add to the robotic data in order to confirm what we had found in the first step and to identify the best combination of clinical and robotic parameters. In this step, we investigated the same groups of variables tested in the first step.

Once we had defined the best data-set of robotic parameters by means of the above steps, we added this to each combination of the clinical parameters selected *a priori*, in order to investigate the predictive power of these pre-selected combinations on adding the robotic parameters.

#### Evaluation of the improvement yielded by combining clinical and robotic parameters

We assessed the improvement in fall-risk prediction on combining both clinical and robotic parameters with respect to the best classifier model that comprised only clinical or only robotic parameters. To do so, we estimated the Net Reclassification Improvement (NRI) and the Integrated Discrimination Improvement (IDI) [43]. The NRI is based on reclassification tables constructed separately for patients with and without events, and quantifies the correct movement in predefined risk categories—upward for events and downward for nonevents. The IDI does not require predefined categories and is the difference between the discrimination slopes of competing models; it can be interpreted as an average over the range of all possible risk cutoffs of the improvements in sensitivity minus the worsening in specificity. A p value <0.05 was considered statistically significant.

NRI and IDI were computed in order to compare the best clinical model or the best robotic model with the best model that combined clinical and robotic parameters.

Robotic raw data were analyzed by means of MATLAB (MathWorks, Natick, MA, USA). Statistical analysis was implemented in Python by means of the Machine Learning scikit-learn library. To calculate the 95% CI of the AUC ROC score by means of the DeLong method [44], the public implementation of the Yandex Data School was used (https://github.com/yandexdataschool/roc_comparison).

## Results

### Participants’ baseline characteristics

During the study period, 100 subjects fulfilled the inclusion criteria and were enrolled in the study. After inclusion, four subjects dropped out from the study owing to personal difficulties in attending the scheduled visits at the hospital center. The final study population therefore consisted of 96 older subjects (34 males and 62 females), with a mean age of 77.17±.49 years.

Baseline characteristics and clinical information on the two groups of subjects (fallers and non-fallers) are summarized in Table 1.

During the one-year follow-up period, 32 participants (33%, 22 female) suffered a fall and were classified as “fallers”; 64 participants (67%, 40 female) did not, and were classified as “non-fallers”. “fallers” were older than “non-fallers” (79.1±6.6 years vs 76.2±6.3 years, mean±SD), more frequently had a history of falls (87.5% vs 62.5%) and performed worse in the walking speed test (0.95±0.22 m/s vs 1.08±0.28 m/s, mean±SD) and in SPPB (7.58±2.55 vs 8.68±237, mean±SD).

As regards robotic evaluation at the baseline, the greatest differences between “fallers” and “non-fallers” were recorded under dynamic conditions (see S3 Table). Specifically, in “fallers”, we observed: a wider range of AP oscillation and a greater oscillation variability (mainly at the trunk level) on the unstable platform (Exercise 4); a wider range of AP and ML oscillation and a greater variability of oscillation on the continuous perturbating platform (Exercise 5); and a wider range of ML tilt on the random perturbating platform (Exercise 6). Additionally, “fallers” showed lower CoP displacement in the forward direction (Exercise 1) than “non-fallers”.

### Feature selection

#### Clinical parameters

On the basis of the literature [9, 11] we selected, from among the clinical parameters collected in the study, the variables commonly used as clinical screening outcomes (SPPB, gait speed, TUG, Tinetti POMA). As we wanted to reproduce the screening procedure generally implemented in clinical practice, we tested all these parameters individually (in addition to the basic data normally collected during visits, such as age, sex and fall history) and then collectively.

#### Robotic parameters

On the basis of the literature [45, 46], previous results and baseline comparison between fallers and non-fallers (see S3 Table), we tested the following sets of parameters: 1) all robotic parameters; 2) a subset of robotic variables based on the results of a previous study [13] (see the selected subset in S2 Table); 3) only dynamic variables (all variables included in exercises 3–7); 4) only static variables (all variables included in exercises 1–2); 5) a selection of variables (see S4 Table) based on significant differences between fallers and non-fallers (S3 Table) and on a detailed analysis of the appropriate literature [47].

#### Clinical and robotic parameters

As reported in the methods section, we evaluated the best combination of clinical and robotic parameters in three steps:

1. Firstly, we fixed the group of robotic variables to those reported in S2 Table. We then found the best combination of clinical variables in terms of ROC AUC score (see Table 2A) by applying the following process: we started by including all the clinical variables (ROC AUC 0.67); from among these, we selected the variables that were significantly different between fallers and non-fallers (SPPB and history of falling), in addition to age and gender (ROC AUC 0.71). We then removed SPPB (ROC AUC 0.70) from this combination and replaced it with “gait speed”, which is one of the items of the SPPB (ROC AUC 0.71); finally, we replaced the continuous variable “gait speed” with the dichotomous variable “low GS” (considering a threshold of 0.8 m/sec on gait speed, ROC AUC 0.73) and removed gender, since this was not significantly different between fallers and non-fallers (ROC AUC 0.73). In this way, we identified the best-performing combination—the combination with the highest ROC AUC and the lowest number of variables—of clinical variables to add to the robotic data-set, which consisted of age, history of falling and low GS (see Table 2A).
2. In the second step, to further improve the performance of the model, we fixed the group of clinical parameters found in the previous step as the best group of clinical parameters (age, history of falling, low GS, Table 2A) and compared different configurations of robotic parameters (see Table 2B). First, we included all robotic parameters (ROC AUC 0.74); then, supported by the literature [45, 46] and the baseline comparison between fallers and non-fallers (see S3 Table) we first excluded the static variables (ROC AUC 0.74) and then included only the static variables (ROC AUC 0.69), thus confirming that the dynamic parameters are more closely correlated with and informative of fall risk. On the basis of the difference between fallers and non-fallers (S3 Table) and of a detailed analysis of the appropriate literature [47], we further reduced the number of variables (ROC AUC 0.80), and found the best data-set of robotic variables (see supplementary results, S4 Table)
3. Finally, having fixed the data-set of robotic parameters to the best data-set identified in the previous step, we conducted a further analysis to test the best data-set of clinical parameters to add to the robotic data. We investigated the same groups of variables tested in the first step, obtaining the results reported in Table 2C and finding the best combinations of clinical and robotic parameters (Table 3C section 1).

**Table 2:** Feature selection.

**A. Feature selection: step 1**	
**Combination of clinical variables**	**Score ROC AUC (computed on adding robotic variables from S2 Table)**
All clinical variables	0.67
Age, Gender, History of falling, SPPB	0.71
Age, Gender, History of falling	0.70
Age, Gender, History of falling, Gait Speed	0.71
Age, Gender, History of falling, low GS	0.73
**Age, History of falling, low GS**	**0.73**
**B. Feature selection: step 2**	
**Combination of robotic variables**	**ROC AUC (computed on adding the best combination of clinical variables from step1)**
All robotic variables	0.74
All dynamic variables (all variables from exercises 3–7)	0.74
All static variables (all variables from exercises 1–2)	0.69
**Dynamic variables (from exercises 3–7) selected from literature**	**0.80**
**C. Feature selection: step 3**	
**Combination of clinical variables**	**ROC AUC (computed on adding the best combination of robotic variables from step 2)**
All clinical variables	0.67
Age, Gender, History of falling, SPPB	0.76
Age, Gender, History of falling	0.76
Age, Gender, History of falling, Gait Speed	0.75
Age, Gender, History of falling, low GS	0.77
**Age, History of falling, low GS**	**0.80**

ROC AUC performance of different combinations of clinical and robotic parameters.

**Table 3:** Comparison of the best fall-risk prediction models.

**A. Combination of clinical parameters, *a priori* selection**						
	**ROC AUC**	**ROC AUC 95% C.I**.	**Mean precision**	**Sensitivity**	**Specificity**	**MCC**
Age, sex, history of falling, SPPB	0.63	0.52–0.75	0.41	0.63	0.66	0.27
Age, sex, history of falling	0.65	0.54–0.77	0.44	0.91	0.39	0.31
**Age, sex, history of falling, TUG**	**0.65**	**0.53–0.77**	**0.45**	**0.72**	**0.65**	**0.35**
Age, sex, history of falling, low GS	0.66	0.55–0.77	0.47	0.75	0.60	0.33
**Age, sex, history of falling, Tinetti POMA**	**0.66**	**0.55–0.78**	**0.45**	**0.81**	**0.56**	**0.36**
**Age, sex, history of falling, TUG, Tinetti POMA, SPPB, low GS**	**0.67**	**0.55–0.79**	**0.56**	**0.69**	**0.65**	**0.32**
Age, sex, history of falling, TUG, Tinetti, SPPB, Low GS, #drugs	0.66	0.53–0.79	0.54	0.69	0.66	0.33
**B. Combination of robotic parameters**						
	**ROC AUC**	**ROC AUC 95% C.I**.	**Mean precision**	**Sensitivity**	**Specificity**	**MCC**
All robotic variables	0.69	0.58–0.80	0.51	0.81	0.56	0.36
Robotic variables selected from previous results (S2 Table)	0,68	0.57–0.79	0,50	0.59	0.74	0.33
All dynamic variables (all variables from exercises 3–7)	0,68	0,58–0.79	0,51	0.78	0.56	0.33
All static variables (all variables from exercises 1–2)	0.48	0.36–0.60	0.32	0.75	0.33	0.09
**Dynamic variables (from exercises 3–7) selected from literature**	**0.73**	**0.63–0.83**	**0.61**	**0.91**	**0.47**	**0.37**
**C. Combination of clinical and robotic parameters**						
	**1. Model selected clinical parameter + robotic parameters (feature selection)**					
	**ROC AUC**	**ROC AUC 95% C.I**.	**Mean precision**	**Sensitivity**	**Specificity**	**MCC**
**Age, history of falling, Low GS**	**0.80**	**0.71–0.89**	**0.68**	***0*.*72***	***0*.*74***	***0*.*44***
	**2. *A priori* selected clinical + robotic parameters**					
Age, sex, history of falling, SPPB	0.76	0.66–0.86	0.56	0.94	0.48	0.42
Age, sex, history of falling	0.76	0.67–0.86	0.57	0.94	0.50	0.43
Age, sex, history of falling, TUG	0.74	0.65–0.84	0.53	0.94	0.48	0.42
Age, sex, history of falling, Low GS	0.77	0.68–0.87	0.58	0.78	0.63	0.39
Age, sex, history of falling, Tinetti	0.75	0.65–0.85	0.56	1.00	0.45	0.47
Age, sex, history of falling, TUG, Tinetti, SPPB, Low GS	0.74	0.63–0.84	0.58	0.56	0.81	0.38
**Age, sex, history of falling, TUG, Tinetti, SPPB, Low GS, #drugs**	**0.81**	**0.72–0.90**	**0.69**	**0.78**	**0.74**	**0.50**

Metrics (ROC AUC, ROC AUC 95% CI, precision, sensitivity, specificity, MCC): results for all the combinations. Section A: combination of clinical parameters determined by a priori selection; Section B: combination of robotic parameters; Section C: combination of clinical and robotic parameters when: 1. Clinical and robotic parameters are determined with model feature selection; 2. Robotic parameters determined by feature selection are added to ‘a priori’ determined clinical parameters (clinical group of section A).

### Prediction models of clinical variables only

**Fig 2:**
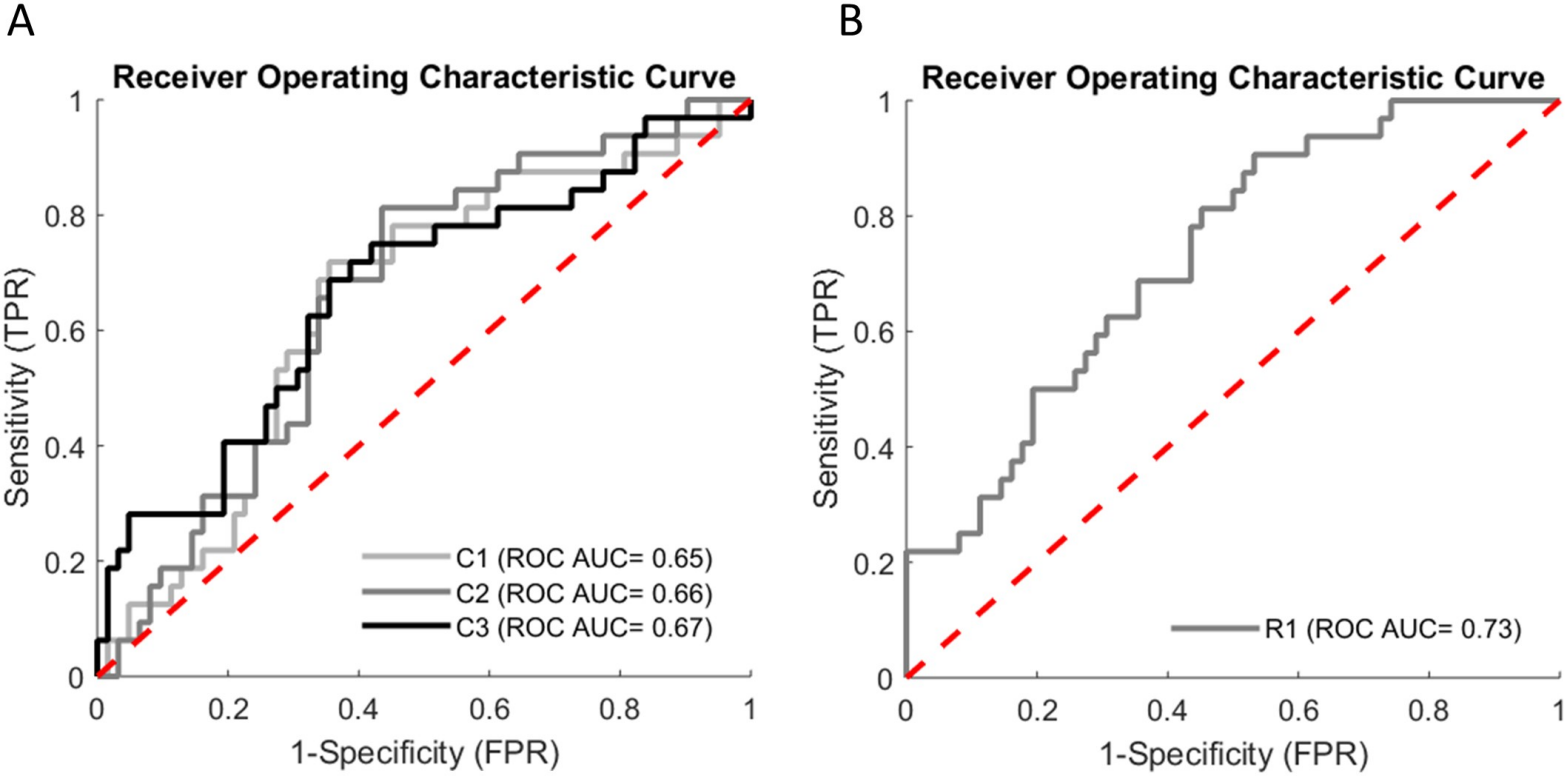
Receiver-operator characteristic (ROC) curves for best models including (A) only clinical and (B) only robotic parameters. **A**. ROC curves obtained from cross-validated fall risk estimate for the best classifier models including only clinical parameters. C = Clinical Group; C1: group including age, sex, history of falling, TUG; C2: group including age, sex, history of falling, Tinetti POMA; C3: group including age, gender, history of falls, TUG, Tinetti POMA, SPPB, and low GS. **B**. ROC curve obtained from cross-validated fall risk estimate for the best classifier model including only robotic parameters. R = Robotic Group. R1: group including 20 selected dynamic variables.

Table 3A shows the accuracy and precision of the different clinical models in predicting falls within 1 year. Of the models that comprised only clinical variables, the one that achieved the highest value of area under the ROC curve (= 0.67, 95% CI 0.55–0.79) consisted of the following parameters: age, gender, history of falls, TUG, Tinetti POMA, SPPB, and low GS (Fig 2A). This model also displayed the highest precision (0.56), one of the highest specificity values (= 0.65), and a moderate MCC score (= 0.32), but lower sensitivity than 4 other combinations. Two of these—the group made up of age, sex, history of falling and TUG (MCC = 0.35) and the one made up of age, sex, history of falling and Tinetti POMA (MCC = 0.36)—presented the highest values of MCC. Both groups displayed higher sensitivity (0.72 and 0.81, respectively) and similar or slightly lower specificity (0.65 and 0.56, respectively), with a slightly lower ROC AUC (0.65 and 0.66, respectively, see Fig 2A).

Notably, no significant differences in ROC AUC score were observed among the functional clinical scales, whether individual or aggregated; indeed, the ROC AUC for different combinations of clinical scales ranged from 0.63 to 0.67. Moreover, the mean precision slightly increased on including a greater number of clinical variables in the model, i.e. it ranged from 0.41 (combination of age, sex, history of falling, SPPB) to 0.56 (on adding TUG, Tinetti POMA, low GS). Conversely, the values of sensitivity and specificity differed for different combinations of variables: specifically, the combination comprising only age, sex and history of falling had the highest sensitivity (0.91) but the lowest specificity (0.39); on adding the Tinetti score, this combination still had a high sensitivity value (0.81), while specificity increased (0.56). For the other combination, sensitivity and specificity ranged from 0.63 to 0.75 and from 0.60 to 0.66, respectively.

The MCC scores of all the groups evaluated were low, ranging from a minimum of 0.27 (weak positive relationship) to a maximum of 0.36 (moderate positive relationship). Regarding the MCC values, the best-performing group was the one that included Tinetti POMA (MCC = 0.36) or TUG (MCC = 0.35) in addition to age, sex and history of falling.

### Prediction models of robotic variables only

Of the model comprising only robotic parameters, the best was the one that included 20 selected dynamic parameters (see S4 Table) (ROC AUC 0.73, 95% CI 0.63–0.83, mean precision 0.6, sensitivity 0.91, specificity 0.47, MCC 0.37, see Table 3B and Fig 2B). This group showed the best ROC AUC score and the highest MCC. The worst performance was found for the group that included only static balance variables, with a ROC AUC score of 0.48 and a very low MCC value (0.09), both indicating very poor predictive power.

### Prediction models of clinical plus robotic variables

**Fig 3:**
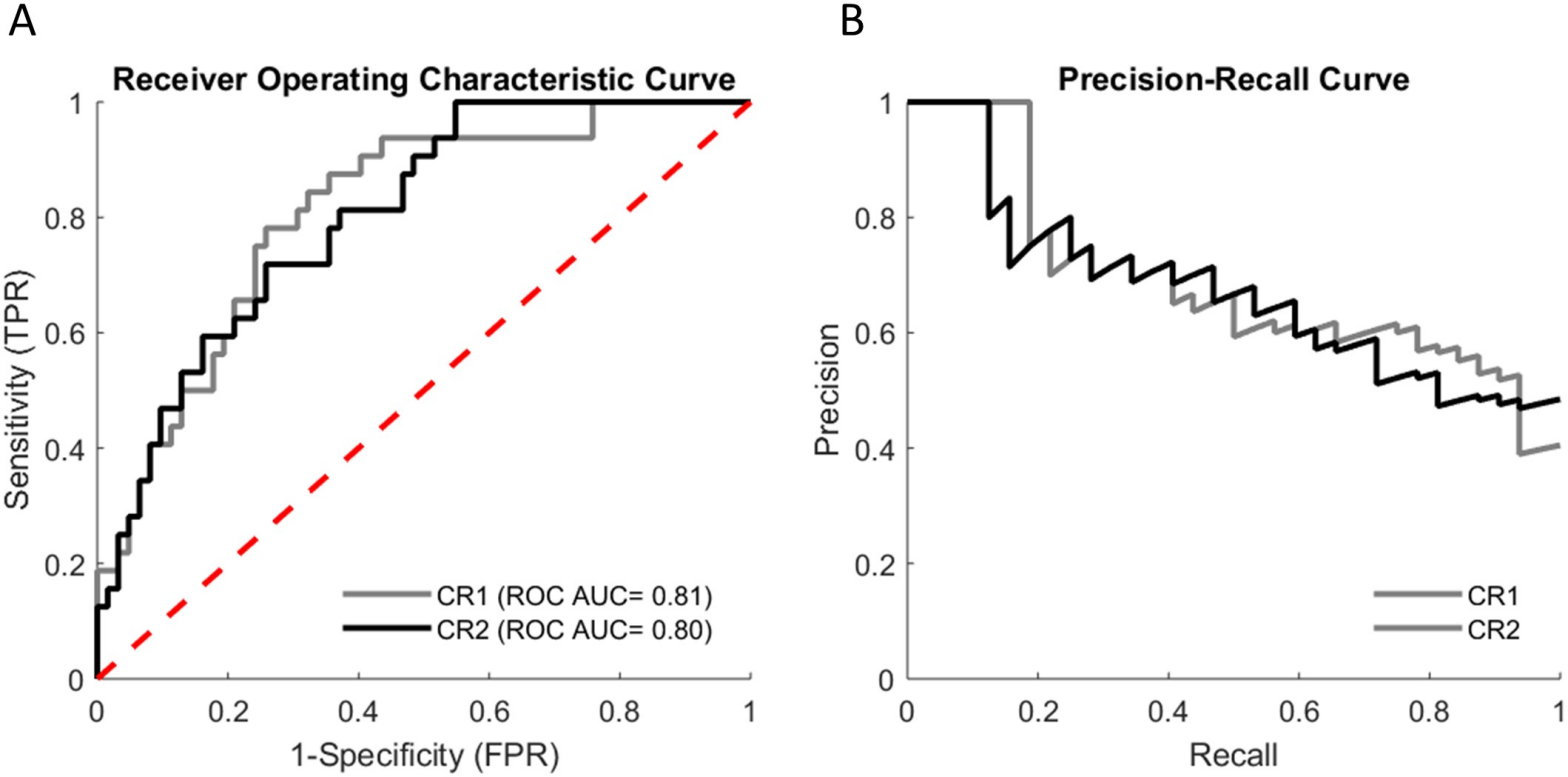
Receiver-operator characteristic (ROC) curve (A) and precision-recall curve (B) of best models including clinical and robotic parameters. ROC curve (A) and precision-recall curve (B) obtained from cross-validated fall risk estimate for the the best classifier models comprising clinical and robotic parameters. CR = Clinical Robotic group. CR1: group including 20 selected robotic parameters plus age, sex, history of falls, number of drugs, TUG, Tinetti POMA, SPPB and low GS; CR2: group comprising 20 selected robotic parameters plus age, history of falls and low GS.

Cross-validated results for the classifier models of fall risk were obtained by combining clinical parameters and 20 robotic variables selected from 46 robotic parameters (S4 Table). The best classifier models that comprised clinical and robotic parameters are described in Table 3, section C (1–2). The classifier model comprising age, sex, history of falls, number of drugs, TUG, Tinetti POMA, SPPB and low GS plus the 20 robotic parameters selected in the feature selection process achieved the best performance score in terms of ROC AUC (ROC AUC of 0.81, 95%CI 0.72–0.90, and a mean precision of 0.69, sensitivity 0.78, specificity 0.74, MCC 0.5, see Fig 3A and 3B). This was also the group with the highest MCC (0.5, strong positive relationship). Notably, the model that included only age, history of falls and low GS, plus the robotic parameters, showed similar accuracy and precision in predicting falls, with slightly lower sensitivity (ROC AUC 0.80, 95% CI: 0.71–0.89; mean precision: 0.68, sensitivity 0.72, specificity 0.74, MCC 0.74) (Fig 3A and 3B). When compared, in terms of ROC AUC, with the best classifier model that comprised only clinical variables (age, gender, history of falls TUG, Tinetti POMA, SPPB, low GS), the two best multifactorial models that comprised both clinical and robotic parameters performed significantly better in predicting fall risk, as demonstrated by the estimated IDI (0.32 (p < 0.001) for the model comprising age, sex, history of falls, number of drugs, TUG, Tinetti POMA, SPPB and low GS plus the 20 robotic parameters, and 0.27 (p<0.001) for the model comprising age, history of falls low GS plus the 20 robotic parameters) and NRI (0.30 (p = 0.02) and 0.31 (p = 0.02), respectively, see Table 4).

**Table 4:** NRI and IDI values for best group comparison.

**A. Best group comparison: clinical vs clinical+robotic**			
**Group: clinical**	**Group: clinical+robotic**	**NRI (p value)**	**IDI (p value)**
Age, sex, history of falling, Tinetti POMA	Age, sex, history of falling, TUG, Tinetti, SPPB, Low GS, #drugs +20 selected robotic variables	0.34 (p = 0.02*)	0.36 (p<0.001*)
Age, sex, history of falling, TUG	Age, sex, history of falling, TUG, Tinetti, SPPB, Low GS, #drugs +20 selected robotic variables*	0.36 (p = 0.01*)	0.38 (p<0.001*)
Age, sex, history of falling, TUG, Tinetti POMA, SPPB, low GS	Age, sex, history of falling, TUG, Tinetti, SPPB, Low GS, #drugs+20 selected robotic variables*	0.30 (p = 0.02*)	0.32 (p<0.001*)
Age, sex, history of falling, Tinetti POMA	Age, history of falling, Low GS +20 selected robotic variables*	0.41 (p = 0.007*)	0.32 (p<0.001*)
Age, sex, history of falling, TUG	Age, history of falling, Low GS +20 selected robotic variables*	0.36 (p = 0.01)	0.33 (p<0.001*)
Age, sex, history of falling, TUG, Tinetti POMA, SPPB, low GS	Age, history of falling, Low GS +20 selected robotic variables*	0.31 (p = 0.02*)	0.27 (p<0.001*)
**B. Best group comparison: robotic vs clinical+robotic**			
**Group: robotic**	**Group: clinical+robotic**	**NRI (p value)**	**IDI (p value)**
Dynamic variables (from exercise 3–7) selected by literature	Age, sex, history of falling, TUG, Tinetti, SPPB, Low GS, #drugs +20 selected robotic variables*	0.15 (p = 0.23)	0.18 (p = 0.003*)
Dynamic variables (from exercise 3–7) selected by literature	Age, history of falling, Low GS+20 selected robotic variables*	0.14 (p = 0.25)	0.13 (p = 0.002*)

NRI and IDI values and their statistical significance, for comparison between A. best groups including only clinical variables vs best groups including clinical and robotic variables B. best groups including only robotic variables vs best groups including clinical and robotic variables. p<0.05 indicates statistical significance;

* indicates the group of 20 robotic variables selected in the feature selection process (S4 Table).

The best models comprising clinical and robotic parameters were also significantly better than the two clinical groups with the highest MCC (those including Tinetti POMA or TUG in addition to age, sex and history of falling, see NRI and IDI values in Table 4). In comparison with the best model comprising only robotic variables, the two best mixed models showed significantly better IDI values, while the NRI value revealed a non-significant change in prediction performance (Table 4).

## Discussion

This prospective study aimed to develop and validate an innovative multifactorial method for fall-risk prediction in older people on the basis of clinical and robotic variables. Our findings demonstrated that the model which best predicted falls during the one-year follow-up period comprised age, sex, history of falls, number of drugs taken and four performance tests (TUG, Tinetti POMA, SPPB and GS) plus 20 parameters measured by the *hunova* robot. The model achieved very good accuracy (ROC AUC of 0.81), showing a significantly better predictive performance than the best predictive model based only on clinical parameters (ROC AUC: 0.81 vs 0.67; mean precision: 0.69 vs 0.56; sensitivity 0.78 vs 0.69; specificity 0.74 vs 0.65) with significant improvement in terms of NRI and, especially, IDI.

Balance is the product of several components, i.e. functional stability limits, underlying motor systems, static stability, reactive postural control, anticipatory postural control, dynamic stability, sensory integration and cognitive influences [10, 25]. The *hunova* robotic assessment evaluated most of these components; stability limits and physiologic standing were evaluated by means of the LOS test (Exercise 1); reactive postural control was evaluated by exercises 5 and 6 on continuous and random perturbating platforms, while dynamic stability and sensory integration were assessed through the “balance on unstable platform” test (Exercise 4).

It is important to highlight that, in the final selection of robotic variables, only parameters coming from dynamic tasks were selected, i.e. dynamic parameters showed higher predictive power than static ones. This result confirms the importance of evaluating dynamic balance tasks instead of only static postural behavior; indeed, our results revealed that, when added to clinical parameters, dynamic variables had the same informative content as static variables, but added further predictive power to the model. This was confirmed when robotic variables were evaluated alone, without any clinical parameters. Indeed, even in this analysis, selected dynamic variables showed the highest performance (ROC AUC 0.73, MCC = 0.37), in contrast with a very poor performance of the group that comprised only static variables (ROC AUC 0.48, MCC = 0.09).

Here, we confirm that the integration of several measures of postural stability can capture the multifactorial nature of fall risk better than a single test [48]. In a previous study, a fall-risk assessment tool that included several clinical variables (falls in the past year, total medications, psychoactive drugs, visual acuity test, touch sensation test, and some balance and performance tests) was better able to distinguish between multiple fallers and non–multiple fallers than the single measures (ROC AUC 0.72, 95% CI = 0.66–0.79) [49]. Interestingly, in that study, the single parameter which achieved the best performance score was a history of falls in the previous year (ROC AUC 0.66 in the development sample, 0.71 in the validation sample) [49].

It is noteworthy that, in the present study, a simplified model made up only of age, history of falls and low GS, in combination with the robotic parameters, showed comparable accuracy (ROC AUC 0.80, 95% CI 0.71–0.89, mean precision 0.68) to that of the model based on all the complete functional and clinical information (i.e. with the additional variables TUG, POMA, SPPB, # of drugs), while displaying only a slight decrease in sensitivity (0.72 vs 0.78). Although this combination presented a lower value of MCC (0.44) than the other best combination (MCC = 0.50), it preserved a significant statistical difference in terms of NRI and IDI when compared with the best combinations of clinical variables. These findings emphasize the role of low GS, which has been defined as the sixth vital sign on account of its ability to represent residual functions and health risks in the elderly [50], and suggest that the parameters measured by the *hunova* robot could integrate and enhance those balance parameters measured by traditional tools.

Interestingly, the combination made up only of robotic parameters performed better than the clinical parameters alone, but worse than the combination comprising both clinical and robotic variables. Specifically, this combination had a higher ROC AUC and mean precision (0.73 and 0.61, respectively) than clinical parameters alone (ROC AUC ranged from 0.63 to 0.67, and precision ranged from 0.41 to 0.56). Moreover, unlike the best combinations of both clinical and robotic parameters, it presented higher sensitivity (0.91) but lower specificity (0.47) at the cut-off point that maximizes the Youden index. This indicates that this combination can detect a higher number of fallers, but that it also includes a higher number of non-fallers in the selection (increasing the number of false positive). Moreover, the NRI regarding the comparison between the best robotic group and the best clinical plus robotic group was not statistically significant, revealing a non-significant difference in prediction.

Most of the models studied achieved an MCC value between 0.30 (= moderate positive relationship) and 0.50 (strong positive relationship), with the highest MCC value being reached by the best group that comprised clinical and robotic parameters and the worst MCC score (= 0.09) being achieved by the model made up only of static robotic parameters. The values obtained for the models that performed best are in line with the MCC values presented in the literature on fall-risk prediction models [51, 52].

Previous studies have applied technology-based devices, such as force platforms, wearable sensors, insoles and floor mats, in order to predict fall risk in community-dwelling older adults [53]. Quantification of movement by means of wearable sensors during the TUG test has proved more accurate in predicting falls than manually-administered TUG and Berg balance test scores (ROC AUC: 0.78, 0.65, 0.62, respectively) [54]. In another study comparing sensor-based fall-risk assessment with conventional assessment tools in a prospective long-term setting, a fall-risk model based on data from accelerometer sensors showed better performance values (ROC-AUC: 0.74, classification accuracy = 72%, sensitivity = 68%, specificity = 74%) than a model derived from a conventional geriatric assessment (classification accuracy = 55%, sensitivity = 63%, specificity = 50%) [33].

More recently, a fall-risk classification model [34] used a neural network to analyze parameters derived from pressure-sensing insoles and tri-axial accelerometers worn at the head, pelvis and left shank; the best-performing model (neural network, dual-task gait data, and input parameters from head, pelvis and left shank accelerometers) showed accuracy = 57%, sensitivity = 43%, and specificity = 65%. However, that study involved only first-time fallers (i.e. retrospective fallers were excluded) and clinical data were not used.

Retrospective studies have recently been performed to identify a fall-risk assessment algorithm that combines technology-based parameters and clinical variables. For example, Bigelow and colleagues identified a logistic regression model that included two traditional sway measures (medial–lateral sway velocity and mean frequency), two fractal measures (both anterior–posterior and medial–lateral short-term α-scaling exponents), and two personal characteristics (BMI and age). Postural parameters were recorded by a force-measuring platform, with participants standing in a comfortable testing condition, with closed eyes. The sensitivity of the model was 75%, and specificity was 94% [55]. A method that automatically combined a fall-risk assessment algorithm based on inertial sensor data and the TUG test with a cross-validated fall-risk assessment based on standard clinical fall-risk factors (gender, height, weight, age, polypharmacy, impaired visual fields and orthostatic hypertension) was proposed by Greene and colleagues: the combined clinical and sensor-based approach yielded a classification accuracy up to 76% (68.5% in an extended cohort), compared with 73.6% for the sensor-based assessment alone, and 68.8% for clinical risk factors alone [56].

To the best of our knowledge, the present study is the first prospective study to yield a classifier model based on clinical variables plus robotic parameters to predict fall risk in community-dwelling older adults.

These findings should be interpreted within the limitations of our study. Firstly, as follow-up was limited to one year, we cannot rule out the possibility that fall-risk patterns may differ over a longer follow-up period. Secondly, owing to the relatively small sample size, we cannot exclude that other clinical and/or functional parameters may significantly influence the accuracy of the predictive model.

As described in the methods section, the problem of the sample size was mainly dealt with by using: i) sparse methods, i.e. Lasso, and ii) a rigorous nested cross-validation approach to improve model stability. Nevertheless, even though the model stability was optimized with respect to the available data, some instability can still be expected and may cause some relevant clinical or robotic parameters to be discarded, and some parameters to be included in the model only owing to noise. This uncertainty is common to all problems involving learning from examples, and can be overcome only by significantly increasing the sample size.

## Conclusion

In conclusion, we propose a multifactorial method of fall-risk assessment that combines functional variables and robotic parameters measured by the *hunova* robot. This approach could be successfully applied in clinical settings to identify people at high risk of falls, with a view to implementing appropriate fall-prevention interventions and generally improving the quality of care.

## Supporting information

S1 Table Robotic parameters. (DOCX)

S2 Table Subset of robotic variable identified from [13]. (DOCX)

S3 Table Baseline values for robotic parameters of the study sample. (DOCX)

S4 Table Robotic parameters selected in the final model. (DOCX)
