# Supplementary material for: Development and validation of a robotic multifactorial fall-risk predictive model: A one-year prospective study in community-dwelling older adults
Source: PLoS One. 2020 Jun 25;15(6):e0234904. doi: 10.1371/journal.pone.0234904 (PMC7316263; doi:10.1371/journal.pone.0234904)
Supplement: S1 Table — (DOCX) [file pone.0234904.s001.docx]

**S1 Table. Robotic parameters**

| **Sensor** | **Parameter** | **Exercise** |
| --- | --- | --- |
| Platform | Max CoP displacement – forward [cm] | Exercise 1 |
|  | Max CoP displacement – backward [cm] | Exercise 1 |
|  | Max CoP displacement – left [cm] | Exercise 1 |
|  | Max CoP displacement – right [cm] | Exercise 1 |
|  | Sway Area [cm^2^] | Exercise 2, 3 (CoP); Exercise 4 (angular displacement) |
|  | Sway Path [cm] | Exercise 2, 3 (CoP); Exercise 4 (angular displacement) |
|  | Range of oscillation -AP [cm] | Exercise 2, 3 (CoP); Exercise 4 (angular displacement) |
|  | Range of oscillation- ML [cm] | Exercise 2, 3 (CoP); Exercise 4 (angular displacement) |
| Trunk | Range of oscillation - AP [deg] | Exercise 2, 3, 4, 5 |
|  | Range of oscillation - ML [deg] | Exercise 2, 3, 4, 5 |
|  | Variability [deg/sec^2^] | Exercise 2, 3, 4, 5 |
|  | Oscillation time- front [s] | Exercise 6 |
|  | Oscillation time- left [s] | Exercise 6 |
|  | Oscillation time – right [s] | Exercise 6 |
|  | Oscillation time – mean [s] | Exercise 6 |
|  | Range tilt ML- front [deg] | Exercise 6 |
|  | Range tilt ML- left [deg] | Exercise 6 |
|  | Range tilt ML- right [deg] | Exercise 6 |
|  | Range tilt ML- mean [deg] | Exercise 6 |
|  | Range tilt AP- front [deg] | Exercise 6 |
|  | Range tilt AP- left [deg] | Exercise 6 |
|  | Range tilt AP- right [deg] | Exercise 6 |
|  | Range tilt AP- mean [deg] | Exercise 6 |
|  | Max tilt ML - mean [deg] | Exercise 6 |
|  | Max tilt AP - mean [deg] | Exercise 6 |
| Platform, Trunk | Total duration (5 repetitions) | Exercise 7 |
|  | Time to stand up and sit down- mean (5 repetitions) | Exercise 7 |
|  | Time to stand up- mean (5 repetitions) | Exercise 7 |
|  | Time to sit down- mean (5 repetitions) | Exercise 7 |
