# Supplementary material for: Development and validation of a robotic multifactorial fall-risk predictive model: A one-year prospective study in community-dwelling older adults
Source: PLoS One. 2020 Jun 25;15(6):e0234904. doi: 10.1371/journal.pone.0234904 (PMC7316263; doi:10.1371/journal.pone.0234904)
Supplement: S2 Table — (DOCX) [file pone.0234904.s002.docx]

**S2 Table**. **Subset of robotic variable identified from [13]**

| **Exercises** | **Variables** |
| --- | --- |
| From exercise 2,3,4,5 | Trunk - Variability [deg/sec^2^] |
|  | Trunk - Range of oscillation - AP [deg] |
|  | Trunk - Range of oscillation - ML [deg] |
| From exercise 6 | Max tilt ML - mean [deg] |
|  | Max tilt AP - mean [deg] |
|  | Range tilt ML- mean [deg] |
|  | Range tilt AP- mean [deg] |
