# Supplementary material for: Development and validation of a robotic multifactorial fall-risk predictive model: A one-year prospective study in community-dwelling older adults
Source: PLoS One. 2020 Jun 25;15(6):e0234904. doi: 10.1371/journal.pone.0234904 (PMC7316263; doi:10.1371/journal.pone.0234904)
Supplement: S3 Table — (DOCX) [file pone.0234904.s003.docx]

**S3 Table. Baseline values for robotic parameters of the study sample (1)**

| **Exercise** | **Sensor** | **Parameter** | **Fallers (n=32)** | **Non fallers (n=64)** | **All subjects (n=96)** | **Significance (p value)** |
| --- | --- | --- | --- | --- | --- | --- |
| **Exercise 1: Limits of stability** | Platform (CoP) | Max CoP displacement – forward [cm] | 5.96±1.31 | 6.70±1.73 | 6.46±1.63 | *0.02** |
|  |  | Max CoP displacement – backward [cm] | 4.52±1.23 | 4.73±1.28 | 4.65±1.26 | *0.43* |
|  |  | Max CoP displacement – left [cm] | 6.75±1.52 | 6.95±1.75 | 6.87±1.66 | *0.56* |
|  |  | Max CoP displacement – right [cm] | 6.79±1.45 | 7.22±1.58 | 7.05±1.55 | *0.10* |
|  |  |  |  |  |  |  |
| **Exercise 2: Balance on static platform, eyes open** | Platform (CoP) | Sway Area [cm^2^] | 2.60±2.68 | 2.86±3.08 | 2.78±2.94 | *0.58* |
|  |  | Sway Path [cm] | 14.26±7.05 | 15.59±7.23 | 15.15±7.16 | *0.24* |
|  |  | Range of oscillation -AP [cm] | 1.65±0.84 | 1.71±0.78 | 1.69±0.80 | *0.58* |
|  |  | Range of oscillation- ML [cm] | 1.61±0.98 | 1.73±1.01 | 1.69±1.00 | *0.42* |
|  | Trunk | Range of oscillation - AP [deg] | 2.66±0.79 | 3.11±1.81 | 2.97±1.57 | *0.27* |
|  |  | Range of oscillation - ML [deg] | 1.66±0.76 | 1.88±1.12 | 1.81±1.02 | *0.54* |
|  |  | Variability [deg/sec^2^] | 0.04±0.01 | 0.04±0.02 | 0.04±0.02 | *0.45* |
|  |  |  |  |  |  |  |
| **Exercise 3: Balance on static platform, eyes closed** | Platform (CoP) | Sway Area [cm^2^] | 5.65±7.07 | 7.58±13.30 | 6.93±11.59 | *0.29* |
|  |  | Sway Path [cm] | 20.90±10.69 | 24.75±14.57 | 23.46±13.47 | *0.16* |
|  |  | Range of oscillation -AP [cm] | 2.72±1.75 | 2.70±1.35 | 2.71±1.49 | *0.50* |
|  |  | Range of oscillation- ML [cm] | 2.19±1.78 | 2.74±2.71 | 2.56±2.44 | *0.22* |
|  | Trunk | Range of oscillation - AP [deg] | 3.31±1.57 | 3.74±2.26 | 3.60±2.06 | *0.36* |
|  |  | Range of oscillation - ML [deg] | 2.03±1.38 | 2.59±3.19 | 2.40±2.74 | *0.69* |
|  |  | Variability [deg/sec^2^] | 0.04±0.02 | 0.05±0.03 | 0.04±0.02 | *0.87* |
|  | | | | | |  |
| **Exercise 4: Balance on unstable platform, eyes open** | Platform (angular displacement) | Sway Area [cm^2^] | 97.37±84.21 | 76.64±67.97 | 82.89±74.00 | *0.16* |
|  |  | Sway Path [cm] | 68.48±36.90 | 56.97±30.43 | 60.48±33.03 | *0.16* |
|  |  | Range of oscillation -AP [cm] | 8.36±3.49 | 6.78±2.81 | 7.27±3.14 | *0.04** |
|  |  | Range of oscillation- ML [cm] | 11.60±5.75 | 9.76±5.75 | 10.32±5.79 | *0.07* |
|  | Trunk | Range of oscillation - AP [deg] | 7.37±4.06 | 5.83±3.72 | 6.29±3.87 | *0.03** |
|  |  | Range of oscillation - ML [deg] | 8.13±5.94 | 6.65±4.23 | 7.10±4.85 | *0.16* |
|  |  | Variability [deg/sec^2^] | 0.09±0.04 | 0.08±0.04 | 0.08±0.04 | *0.04** |

Values are expressed as mean ± standard deviation; AP= anteroposterior; ML= mediolateral; significance was tested with a Mann Whitney U test; *p>0.05;

**S3 Table. Baseline values for robotic parameters of the study sample (2)**

| **Exercise** | **Sensor** | **Parameter** | **Fallers (n=32)** | **Non fallers (n=64)** | **All subjects (n=96)** | **Significance (p value)** |
| --- | --- | --- | --- | --- | --- | --- |
| **Exercise 5: Balance on** **continuous perturbating platform with eyes open** | Trunk | Range of oscillation - AP [deg] | 9.57±4.18 | 8.15±5.32 | 8.57±4.99 | *0.02** |
|  |  | Range of oscillation - ML [deg] | 8.00±3.22 | 7.64±6.22 | 7.74±5.39 | *0.03** |
|  |  | Variability [deg/sec^2^] | 0.12±0.04 | 0.10±0.05 | 0.10±0.05 | *0.006** |
|  | | | | | |  |
| **Exercise 6: Balance on** **random perturbating platform with eyes open** | Trunk | Oscillation time- front [s] | 1.18±0.68 | 1.06±0.70 | 1.09±0.69 | *0.22* |
|  |  | Oscillation time- left [s] | 1.54±0.86 | 1.54±0.96 | 1.54±0.92 | *0.78* |
|  |  | Oscillation time – right [s] | 2.32±1.23 | 2.33±1.38 | 2.33±1.32 | *0.87* |
|  |  | Oscillation time – mean [s] | 1.68±0.89 | 1.64±0.97 | 1.65±0.93 | *0.71* |
|  |  | Range tilt ML- front [deg] | 3.66±1.96 | 3.18±1.74 | 3.33±1.81 | *0.15* |
|  |  | Range tilt ML- left [deg] | 9.85±2.53 | 8.76±2.89 | 9.10±2.80 | *0.08* |
|  |  | Range tilt ML- right [deg] | 9.92±2.72 | 8.70±3.21 | 9.07±3.08 | *0.03** |
|  |  | Range tilt ML- mean [deg] | 7.81±1.80 | 6.88±2.22 | 7.17±2.12 | *0.03** |
|  |  | Max tilt ML- mean [deg] | 5.60±1.41 | 4.81±1.57 | 5.06±1.55 | *0.01** |
|  |  | Range tilt AP- front [deg] | 11.28±4.11 | 10.76±5.23 | 10.88±4.88 | *0.41* |
|  |  | Range tilt AP- left [deg] | 5.10±3.33 | 5.29±3.35 | 5.24±3.31 | *0.61* |
|  |  | Range tilt AP- right [deg] | 4.55±2.86 | 4.67±3.06 | 4.62±2.97 | *0.93* |
|  |  | Range tilt AP- mean [deg] | 6.98±2.91 | 6.91±3.38 | 6.91±3.21 | *0.82* |
|  |  | Max tilt AP- mean [deg] | 5.52±2.27 | 5.33±2.53 | 5.38±2.43 | *0.73* |
|  | | | | | |  |
| **Exercise 7: Five times sit to stand** |  | Total duration (5 repetitions) | 18.13±4.98 | 19.05±6.67 | 18.73±6.16 | *0.64* |
|  |  | Time to stand up and sit down- mean (5 repetitions) | 3.54±1.07 | 3.79±1.43 | 3.72±1.32 | *0.47* |
|  |  | Time to stand up- mean (5 repetitions) | 1.72±0.59 | 1.70±0.78 | 1.71±0.72 | *0.98* |
|  |  | Time to sit down- mean (5 repetitions) | 1.82±0.60 | 2.09±0.86 | 2.00±0.79 | *0.19* |

Values are expressed as mean ± standard deviation; AP= anteroposterior; ML= mediolateral; significance was tested with a Mann Whitney U test; *p>0.05;
