# Supplementary material for: Development and validation of a robotic multifactorial fall-risk predictive model: A one-year prospective study in community-dwelling older adults
Source: PLoS One. 2020 Jun 25;15(6):e0234904. doi: 10.1371/journal.pone.0234904 (PMC7316263; doi:10.1371/journal.pone.0234904)
Supplement: S4 Table — (DOCX) [file pone.0234904.s004.docx]

**S4 Table. Robotic parameters selected in the final model**

| **Exercise** | **Variable** |
| --- | --- |
| **Exercise 1: Limits of stability** | Max CoP displacement – forward [cm] |
|  | Max CoP displacement – backward [cm] |
|  | Max CoP displacement – left [cm] |
|  | Max CoP displacement – right [cm] |
|  |  |
| **Exercise 4: Balance on unstable platform, eyes open** | Sway Path [cm] |
|  | Range of oscillation -AP [cm] |
|  | Range of oscillation- ML [cm] |
|  | Trunk-Variability [deg/sec^2^] |
|  |  |
| **Exercise 5: Balance on continuous perturbating platform with eyes open** | Trunk- Range of oscillation - AP [deg] |
|  | Trunk- Range of oscillation - ML [deg] |
|  | Trunk- Variability [deg/sec^2^] |
|  |  |
| **Exercise 6: Balance on random perturbating platform with eyes open** | Trunk- Oscillation time- front [s] |
|  | Trunk- Oscillation time- left [s] |
|  | Trunk- Oscillation time – right [s] |
|  | Trunk- Range tilt ML- left [deg] |
|  | Trunk- Range tilt ML- right [deg] |
|  | Trunk- Range tilt AP- left [deg] |
|  | Trunk- Range tilt AP- right [deg] |
|  |  |
| **Exercise 7: Five times sit to stand** | Time to stand up and sit down- mean (5 repetitions) |
|  | Time to sit down- mean (5 repetitions) |
